# The Phylogenetic Limits to Diversity-Dependent Diversification

**DOI:** 10.1093/sysbio/syac074

**Published:** 2022-12-01

**Authors:** Rampal S Etienne, Bart Haegeman, Álvaro Dugo-Cota, Carles Vilà, Alejandro Gonzalez-Voyer, Luis Valente

**Affiliations:** Groningen Institute for Evolutionary Life Sciences, University of Groningen, Box 11103, 9700 CC Groningen, The Netherlands; CNRS/Sorbonne Université, UMR7621 Laboratoire d'Océanographie Microbienne, 1 av. Pierre Fabre, 66650 Banyuls-sur-Mer, France; Conservation and Evolutionary Genetics Group, Doñana Biological Station (EBD-CSIC), 41092 Seville, Spain; Conservation and Evolutionary Genetics Group, Doñana Biological Station (EBD-CSIC), 41092 Seville, Spain; Department of Evolutionary Ecology, Instituto de Ecología, Universidad Nacional Autónoma de México, 04510 Mexico City, Mexico; Groningen Institute for Evolutionary Life Sciences, University of Groningen, Box 11103, 9700 CC Groningen, The Netherlands; Naturalis Biodiversity Center, Darwinweg 2, 2333 CR Leiden, The Netherlands

## Abstract

While the theory of micro-evolution by natural selection assigns a crucial role to competition, its role in macroevolution is less clear. Phylogenetic evidence for a decelerating accumulation of lineages suggests a feedback of lineage diversity on diversification. However, does this feedback only occur between close relatives, or do distant relatives also influence each other’s diversification? In other words: are there phylogenetic limits to this diversity-dependence? Islands form ideal systems to answer these questions because their boundedness facilitates an overview of all potential competitors. The DAISIE (Dynamic Assembly of Island biota through Speciation Immigration and Extinction) framework allows for testing the presence of diversity-dependence on islands given phylogenetic data on colonization and branching times. The current inference models in DAISIE assume that this diversity-dependence only applies within a colonizing clade, i.e., all mainland species can colonize and diversify independently from one another. We term this clade-specific (CS) diversity-dependence. Here we introduce a new DAISIE model that assumes that diversity-dependence applies to all island species of a taxonomic group regardless of their mainland ancestry, i.e., diversity-dependence applies both to species within the same clade and between different clades established by different mainland species. We call this island-wide (IW) diversity-dependence. We present a method to compute a likelihood for this model given phylogenetic data on colonization and branching events and use likelihood ratio bootstrapping to compare it to the likelihood of the CS model in order to overcome biases known for standard model selection. We apply it to the diversification of *Eleutherodactylus* frogs on Hispaniola. Across the Greater Antilles archipelago, this radiation shows repeated patterns of diversification in ecotypes that are similar across clades. This could be suggestive of overlapping niche space and hence between-clade interactions, i.e., IW diversity-dependence. But it could also be suggestive of only within-clade interactions because between-clade interactions would have blocked the same ecotype from re-appearing. We find that the CS model fits the data much better than the IW model, indicating that different colonizations while resulting in similar ecotypes, are sufficiently distinct to avoid interacting strongly. We argue that non-overlapping distributions between clades (both spatially and in terms of ecotypes) cannot be used as evidence of CS diversity-dependence, because this pattern may be a consequence of IW diversity-dependence. By contrast, by using phylogenetic data rather than distributional data our method does allow for inferring the phylogenetic limits to diversity-dependent diversification. We discuss possibilities for future extensions and applications of our modelling approach. [Adaptive radiation; birth-death model; Caribbean; diversity-dependence; Eleutherodactylus; island biogeography.]

“As species of the same genus have usually, though by no means invariably, some similarity in habits and constitution, and always in structure, the struggle will generally be more severe between species of the same genus, when they come into competition with each other, than between species of distinct genera”. This statement by Darwin in the Origin of Species (Darwin, 1859), known as the competition-relatedness hypothesis (Cahill et al., 2008) or the phylogenetic limiting similarity hypothesis (Violle et al., 2011), or Darwin’s naturalization hypothesis in the field of invasion biology (Proches et al., 2008), has been the subject of debate over the past decades, particularly in the field of phylogenetic community ecology (Mayfield and Levine, 2010; HilleRisLambers et al., 2012; Gerhold et al., 2015; Narwani et al., 2015; Pigot and Etienne, 2015; Germain et al., 2016; Cadotte et al., 2017; Wilcox et al., 2018). The consequences of the competition-relatedness hypothesis for macroevolution have received much less attention. Darwin (1859) formulated these consequences himself as “each new variety or species, during the progress of its formation, will generally press hardest on its nearest kindred, and tend to exterminate them.” This implies that with increasing diversity, speciation rates decline or extinction rates increase. This phenomenon has been referred to as diversity-dependent diversification (also somewhat confusingly called density-dependent diversification) since the 1970s (Raup et al., 1973; Walker and Valentine, 1984). Rabosky (2013) distinguishes Darwinian diversity-dependence, which does not imply an upper bound, from asymptotic diversity-dependence, which by definition does impose an upper bound on diversity. We leave the question aside whether an upper bound exists, and rather focus on the commonality of these types of diversity-dependence: that diversity levels affect diversification, and in particular colonization and speciation rates decline with increasing diversity. (In other words: even though in this paper we model this negative diversity-dependence with a finite upper bound, we do not believe that whether the diversity limit is finite or infinite is crucial for our findings.)

There have been many suggestions of how such diversity limits come about (Rabosky, 2013; Rabosky and Hurlbert, 2015). Here we do not enter this discussion, but we are interested in whether there is a phylogenetic limit to the effect of diversity, i.e., whether diversity-dependence only acts between closely related species and/or between distantly related species. There is considerable support for diversity-dependence in clades of phylogenetically closely related species (Foote and Miller, 2006; Phillimore and Price, 2008; Rabosky and Glor, 2010; Etienne et al., 2012; Jønsson et al., 2012; Foote et al., 2018), but there is also some evidence that phylogenetically distantly related (but ecologically similar) taxa reduce each other’s diversification rates (Stanley, 1973; Sepkoski, 1996; Valkenburgh, 1999; Jablonski, 2008; Silvestro et al., 2015; Pires et al., 2017). However, the latter evidence is relatively scarce and comes mostly from fossil data. The question then presents itself whether molecular phylogenies can also inform us about the phylogenetic limits to diversity-dependent diversification.

We propose that islands are the ideal arena to study these questions because they are clearly defined systems where (exceptional) radiations have occurred. Moreover, as islands tend to be depauperate, we see cases where species released from the competition have radiated to fill niches usually occupied by a different clade, e.g., woodpecker finches in the Galápagos. In MacArthur and Wilson’s original work on island biogeography (MacArhur and Wilson, 1967) speciation receives little attention, and therefore the same applies to diversity-dependent speciation, but they assume that colonization and extinction are diversity-dependent, as per capita colonization rates decrease and per capita extinction rates increase with increasing island diversity. The General Dynamic Model of island biogeography (Whittaker et al., 2008) explicitly assumes that the island’s carrying capacity influences the diversification rates. However, neither of these classic works discusses the phylogenetic nature of the limits to diversification. Here, we consider two types of diversity-dependence, differing in the phylogenetic extent of diversity-dependence: the clade-specific (CS) level, where only species that descend from the same mainland (extinct or extant) species (possibly through multiple colonizations) reduce each other’s speciation rate and colonization rates, and the island-wide (IW) level, where all island species of a predefined taxonomic group, that may descend from very different mainland ancestors, inhibit each other’s speciation and colonization (see Fig. 1). Our focus is on species that occur on the island, and therefore by ‘clade’ we refer to a lineage of island species descending from the same mainland ancestor species. These island lineages are evidently embedded in a wider lineage containing both insular and mainland species, but the phylogeny of the species outside of the island is not considered here. The CS scenario can be modelled by assuming a carrying capacity or upper limit to the number of species for each clade, while the IW model can be modelled by assuming an island-wide carrying capacity or upper limit to the total number of species. The CS and IW models are thus two extremes of a continuum of diversity effects on colonization and speciation. In practice, these effects will not stop directly at the clade established by a colonizing species as in the CS model, but they will also not generally extend to all island species of the considered taxon as in the IW model. Modelling the continuum between the extremes taking into account the phylogenetic (or phenotypic) distances between the mainland species would be ideal, but the IW model is already difficult to handle mathematically and computationally (see below), and thus modelling an intermediate case is currently unfeasible. However, we believe that with these two extremes we are still able to gain more insight into the phylogenetic limits to diversity-dependent colonization and speciation.

**Figure 1 .**
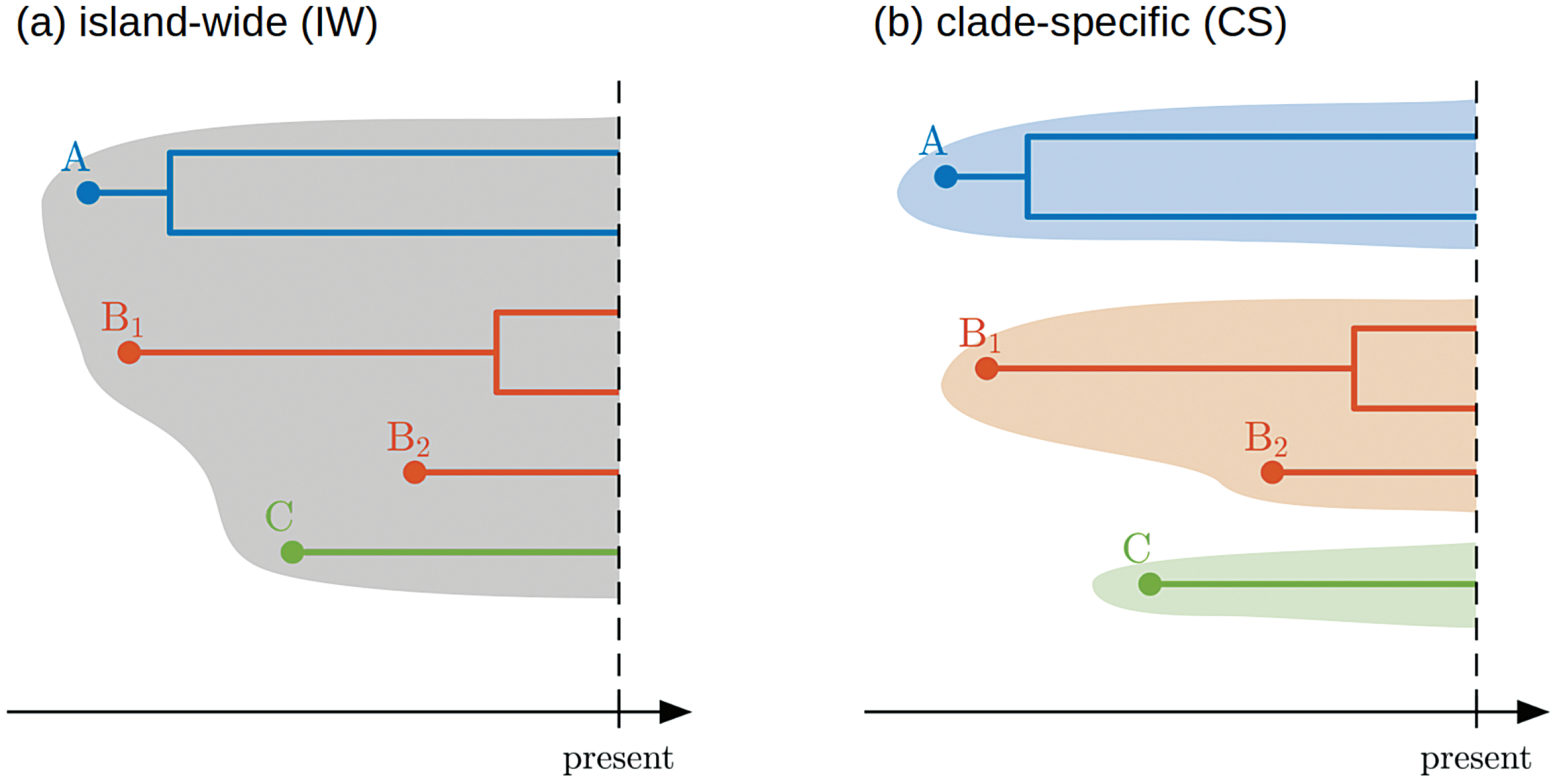
Schematic representation of the two types of diversity-dependence. Phylogenetic trees A to C represent clades of island species descending from different colonization events. In the IW model (a) diversity-dependence extends to all island lineages in the data, whereas in the CS model (b) diversity-dependence extends only to species descending from the same mainland species. In this example there is a recolonization of species B. In the CS model diversification in clade $B_{1}$ depends on diversity in clade $B_{1}$ as well as clade $B_{2}$, because these two clades descend from the same mainland ancestor (in a phylogeny that includes exclusively the island species, clades $B_{1}$ and $B_{2}$ would form a single monophyletic lineage). This is evidently also true for the IW model. For the CS model the same clade-level carrying capacity K applies to each clade separately (represented by the extent of each shaded area), while for the IW model it applies to all clades together.

Diversity-dependence in speciation rates and colonization rates has been incorporated in the DAISIE framework (Dynamic Assembly of Island biota through Speciation, Extinction, and Immigration, Valente et al. (2015)) that allows maximum likelihood estimation of rates of colonization, speciation and extinction from phylogenetic data of the clades that colonized an island (or archipelago). In the first simulations in this framework, diversity-dependence was of the IW-type (Valente et al., 2014). For inference (i.e., parameter estimation), only the CS model was implemented (Valente et al., 2015), using insight from analyses on single clades of closely-related species (Rabosky and Lovette, 2008; Etienne et al., 2012), because the IW model presented technical difficulties. Here we overcome (some of) these technical difficulties by presenting a method to compute the likelihood of colonization and branching events under the IW model.

We illustrate our method with an application to the colonization of Hispaniola by five lineages of *Eleutherodactylus* frogs (genus *Eleutherodactylus*; Dugo-Cota et al., 2019), for which both CS and IW models can be verbally argued to apply. On the one hand, these lineages show, across the Greater Antillean archipelago, repeated patterns of diversification into a similar set of ecotypes (Dugo-Cota et al., 2019), suggesting a limited set of niches is available, which in turn implies that diversity-dependence acts, but no further than within each clade (CS). On the other hand, the relatively low geographic overlap in ecotypes between clades on Hispaniola suggests that diversity-dependence extends to all *Eleutherodactylus* species on the island (IW) because species may have blocked colonization of the same ecotype regardless of their phylogenetic relatedness. Our analysis, using only phylogenetic data, shows that the CS model fits the data much better than the IW model. We discuss this result and provide suggestions for further research avenues.

## METHODS

Under the original DAISIE inference model (Valente et al., 2015) and its subsequent extensions (Valente et al., 2017a; [Bibr CIT0049][Bibr CIT0050]) species can colonize an island at a rate γ, go extinct at a rate μ, and speciate via cladogenesis (when one island species splits into two, forming two new endemic species) at a rate $\lambda^{c}$ or via anagenesis (when one island species diverges from its mainland ancestor becoming a new endemic species, without leading to an increase in diversity on the island) at a rate $\lambda^{a}$. CS-type diversity-dependence is implemented by allowing for rates of cladogenesis and colonization to decline with increasing diversity within a clade, with the number of species within each clade being limited by a CS carrying capacity, K. The maximum-likelihood implementation of DAISIE allows γ, μ, $\lambda^{c}$, $\lambda^{a}$, and K to be estimated based on the distribution of times of island colonization and branching times within an island, extracted from divergence-dated molecular phylogenies. A diversity-independent model (DI) is also implemented, i.e., by fixing K to infinity so that $\lambda^{c}$ and γ do not decline with diversity.

A logical alternative model to CS in the island context is the IW model, where instead of a K per clade there is an island-wide K that determines the maximum number of species that can coexist on an island across all clades. This model was implemented in the first version of DAISIE, but only in simulations (Valente et al., 2014). Until now, estimating the parameters of an IW model has not been attempted, because (i) the model equations are rather cumbersome to write down and implement, and (ii) parameter estimation is computationally demanding in terms of memory requirements and runtimes, even for small data sets, because the likelihood computation requires solving a large number of ordinary differential equations, see Supplementary Material. Here we take on these hurdles. We develop a method of estimating parameters of an IW model from phylogenetic data. The data requirements, parameters, and simulation approach of the DAISIE IW model are the same as for CS, except that diversity-dependence in $\lambda^{c}$ and γ is determined by an island-wide K, so that these rates decline with a diversity of all island species rather than simply diversity of the colonist clade they belong to.

### Likelihood of Colonization and Branching Data for the IW Model

We compute the likelihood of the data, consisting of colonization and branching events, for the IW model using the Q approach (Etienne et al., 2012; Laudanno et al., 2019). This approach is named after the quantity Q(t), which is the probability that a random realization of the model is consistent with the data up to an arbitrary time t. In the Supplementary Material, we construct the differential equations governing the dynamics of Q(t), and explain how these equations which apply to the dynamics between colonization and branching events, are connected to one another across the colonization and branching events. By solving these equations from the island emergence time to the present, we obtain $Q(t_{p})$, the quantity Q(t) evaluated at the present time $t_{p}$, from which the likelihood can be extracted (see Supplementary Material for details).

Our computational procedure is based on the assumption that we have full information about the extant species. That is, we assume that the island phylogenies of the full set of extant species are known, together with the corresponding colonization times. This assumption simplifies not only the likelihood computation but also the comparability with the CS likelihood. Indeed, in the case of partial sampling from the phylogeny, the CS model distinguishes to what clade the missing species belong, while the IW model does not, making their likelihood incomparable. To guarantee full comparability we have also treated the likelihood of the CS model as a product of IW likelihoods with a mainland pool size of 1 across the M mainland species. That is, CS and IW only differ in whether clades established by mainland ancestors are independent (CS) or are connected through each other’s diversity (IW).

### Model Fitting

We fitted five DAISIE models: a model without diversity-dependence (DI, four free parameters), a model with clade-specific diversity-dependence (CS-DD, five free parameters), a model identical to CS-DD but without anagenesis (CS-DD-noA, four free parameters); a model with island-wide diversity-dependence (IW-DD, five free parameters), and a model identical to IW-DD but without anagenesis (IW-DD-noA, four free parameters). In all DD models the per capita rates of cladogenesis $\lambda_{N}^{c}$ and colonization $\gamma_{N}$ were assumed to linearly decline with diversity:


$$\begin{array}{l} \begin{array}{ll} & \lambda_{N}^{c}=\lambda_{0}^{c}(1-N/K)\\ & \gamma_{N}=\gamma_{0}(1-N/K) \end{array} \end{array}$$

where N is the total number of species in a clade in the CS model, and the total number of species on the entire island in the IW model. K is the carrying capacity per clade for the CS model (hence the same for each clade) and for the entire island for the IW model. We follow the original DAISIE model (Valente et al., 2015) by assuming no diversity-dependence in extinction or anagenesis, and we solely focus on diversity-dependence in rates of colonization and cladogenesis.

### Phylogenetic Data

We used the dated phylogeny of *Eleutherodactylus* frogs by Dugo-Cota et al. (2019), which is based on four mitochondrial and three nuclear genes. The data set comprises 152 species of the genus, including 148 Caribbean species, i.e., 89% of the Caribbean diversity, as well as four continental species. The divergence-dated phylogeny was reconstructed in BEAST v1.8.2, using secondary time calibration points extracted from the wider eleutherodactyline phylogeny of Heinicke et al. (2007), Dugo-Cota et al. (2019) reconstructed the biogeographical history of Caribbean *Eleutherodactylus* using BioGEOBEARS (Matzke, 2013, 2014) with a time-stratified analysis and nine geographical regions. They inferred five colonization of Hispaniola from the mainland and surrounding islands (which are collectively referred to as the mainland hereafter), each of which radiated on the island, to a great or lesser degree, producing five in situ radiations of 28, 21, 8, 5, and 3 species (Table 1).

**Table 1.** Characteristics of the five clades on Hispaniola: diversity, colonization times (with uncertainty range) and geographical distribution within the island of Hispaniola

Clade	Number of species	Colonization time (Ma)	Geographical distribution
1	28	22.09 (18.21–26.36)	Mixed
2	21	13.75 (10.73–16.97)	100% South
3	3	11.03 (8.31–13.89)	100% North
4	5	8.85 (6.91–10.8)	100% South
5	8	8.43 (6.3–10.73)	87.5% North

The Dugo-Cota et al. (2019) phylogeny includes 57 of the 66 Hispaniola species. Because fitting the IW model assumes complete sampling of the extant species of the focal island, we inserted the missing Hispaniola species by assigning them to random locations within the Hispaniola subclades that they have been hypothesized to belong to. Information on the nine missing species and the detailed rationale for including them in a given subclade are given in Supplementary Table S1. There is no genetic data available on GenBank for these missing species because they have been recently described, are known from a single specimen, or are possibly extinct. We used a set of functions from the phytools R package to assign missing species to clades (Revell, 2012). Four of the missing species were previously considered subspecies, and have recently been elevated to species, and we thus randomly inserted them at any height along the tip branch of the species they were previously assigned to. Four other species have been proposed to belong to well-defined terminal clades based on morphology, and we randomly inserted them at any position and at any height within those clades. We repeated this procedure 100 times on the five clades from the maximum clade credibility tree from BEAST, producing 100 sets of five clades with complete sampling. The exact procedure is detailed in Supplementary Table S1. One of the missing species, *E. neiba* was not added to the tree because there is no previous hypothesis regarding its phylogenetic position. We ran a sensitivity analysis including this species as a separate colonization, to assess whether in the unlikely case it formed a separate clade this would affect the results. These analyses showed that even if *E. neiba* formed an independent colonization, the same model would still be preferred. We therefore did not include it in the analyses, as it is unlikely to modify the main findings.

**Figure 2.**
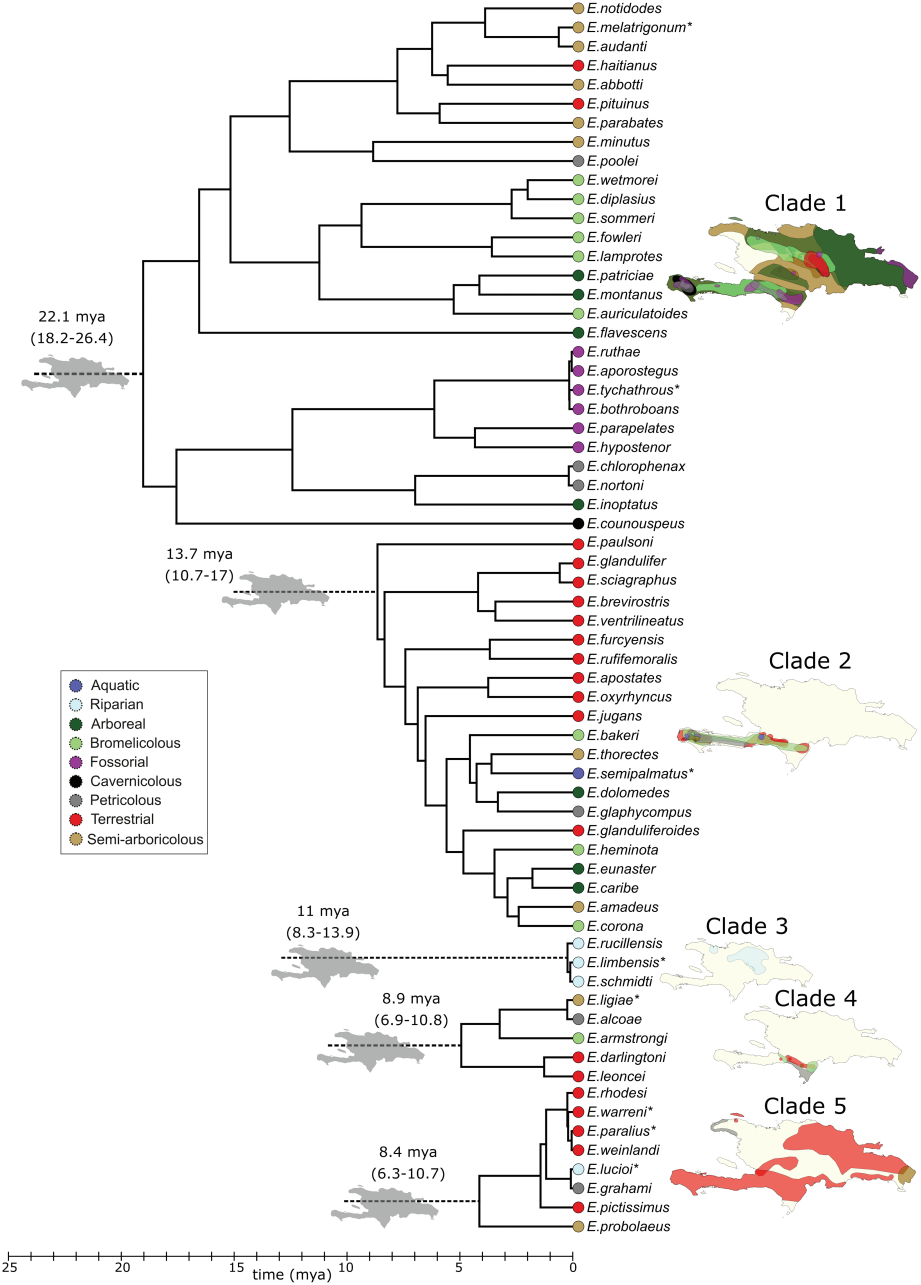
Phylogeny of the Hispaniola *Eleutherodactylus* frogs, and the ecotypes and spatial distribution of the tip species. A separate time-calibrated phylogenetic tree is shown for each of the five clades. Gray islands show the five inferred independent colonization events of Hispaniola. Colors at the tips of the phylogeny represent the species ecotypes (see legend). The same colors are used to show the species distributions for each independent colonization on the Hispaniola map (for visual clarity some transparency has been applied). The asterisk indicates where missing species have been added to the phylogeny according to taxonomic information, see Supplementary Table S1. We note that our inference method only uses the phylogenetic information in the data, i.e., only the colonization and branching events.

We extracted colonization and branching times for each of the five Hispaniola radiations from these data. Colonization times were assumed to be the stem ages of the Hispaniola clades, as the stem age marks the divergence from the mainland sister clade (which is assumed to be due to the colonization event). Information on each of the Hispaniola clades is given in Table 1 and the phylogeny is shown in Figure 2.

As the downstream DAISIE analyses are computationally demanding, we wanted to use only one data set for subsequent analyses. To perform an informed selection of the tree, we fitted the CS and the IW model with no anagenesis to each of the 100 sets of trees. The results of the analysis on the 100 sets of trees are shown in Supplementary Table S2. The preferred model in all trees was CS. We thus used only tree set 52, which was the one with the highest likelihood for CS, for all subsequent analyses (hereafter “empirical data set”). This may seem to introduce a bias in favor of the CS model, but we note that all loglikelihood differences (and all parameter estimates) were very similar across the 100 sets of trees: the loglikelihood differences between CS and IW were between 5.2 and 6.6 with a median of 5.9 and set 52 had a loglikelihood difference of 6.4. All 100 data sets would have led to the same conclusions in our model comparison (see Results). All sets of trees and corresponding DAISIE colonization/branching time R objects are provided in the Supplementary Material. The simulation code functions to compute the likelihood under the two models, and a tutorial on how to run simulations and perform model-fitting are available in the R package DAISIE on CRAN and on Github (https://github.com/rsetienne/DAISIE).

### Likelihood Optimization on the Empirical Data

We fitted each of the five DAISIE models five times to the empirical data set using different random sets of starting parameters to avoid being trapped in local likelihood optima. We assumed an island age of 30 million years, consistent with the paleogeographical reconstruction of Iturralde-Vinent (2006) for when Hispaniola was isolated from other landmasses. The mainland pool size M was set to 1000 frog species. We note that this value is not crucial, because mainland pool size affects only the rate of colonization; the product of mainland pool size and the rate of colonization, i.e., the total rate of colonization, is practically constant (Valente et al., 2019). Indeed, parameter estimates were very similar for optimizations with M=300.

Maximum likelihood optimizations were run on the high-performance (Peregrine) cluster of the University of Groningen. Optimization of DI and CS-type models generally converged in a few hours. IW-DD model optimizations took between a few hours to 10 days to complete.

### Goodness-of-Fit

We simulated 5000 data sets using the maximum likelihood parameters of the preferred CS-type model (CS–DD no anagenesis) and preferred IW-type model (IW–DD no anagenesis), hereafter the CS and IW models. We then plotted relevant statistics from the simulated data sets and compared them to those in the empirical data to study how well the models fit the data.

### Bootstrap Analysis

We computed the AIC and BIC values and weights for model comparison, but because model selection involving diversity-dependent models is known to be troublesome (Etienne et al., 2016), we used a parametric bootstrap likelihood ratio test similar to Boettiger et al. (2012). This bootstrap analysis additionally allowed us to assess the bias and precision of parameter estimates. We chose the first 1000 out of the 5000 data sets from each of the CS and IW simulations. Not all 5000 simulated data sets were used for the bootstrap likelihood ratio test, because the subsequent analyses on these data sets were computationally demanding. For each of the chosen 2000 data sets, we fitted both CS and IW models, resulting in a total of 4000 maximum likelihood optimizations. As starting values of the optimizations we used the maximum likelihood parameters of the given model obtained for the empirical data, to make it as likely as possible that we will find the global likelihood optimum, as it is expected to be around these empirical maximum likelihood parameters. To really ensure local optima are avoided, the optimizations would need to be run many times from many different starting values, but this was computationally unfeasible. In some cases, it was not possible to use the parameters obtained in the optimization analyses as initial parameters for the optimization. For instance, if a clade in data sets generated under the IW model had more species than the value of K estimated for the CS model fitted to the empirical data, using that K as a starting value to fit the CS model to the IW-simulated data would give a likelihood of 0. Therefore we calculated the starting K for each data set using the largest value of either the K estimated from the empirical data for the given model being fitted, or the maximum number of species in a clade (CS model) or the total number of species on the island (IW model) in the simulated data set.

For the bootstrap likelihood ratio test, we compared the logarithm of the likelihood ratio of CS and IW in the empirical data (i.e., loglikelihood difference, loglikelihood of the CS model_–_loglikelihood of the IW model) with the distribution of the logarithm of likelihood ratios from the data sets simulated under CS and under IW (1000 data sets each). We computed the 95th percentile of the distribution under the IW model. If the loglikelihood difference of the empirical data falls to the right of this value, then they are unlikely to be produced by the IW model, and if it is well within the distribution of the data generated under the CS model, the CS model is selected. We also computed the 5th percentile of the distribution under the CS model. If the loglikelihood difference of the empirical data falls to the left of this value, then they are unlikely to be produced by the CS model, and if it is well within the distribution for the data generated under the IW model, the IW model is selected. If the loglikelihood difference of the empirical data falls between the two percentiles, then no model can be selected decisively.

## RESULTS

### Likelihood Optimization on the Empirical Data

Convergence of the five independent optimizations per model to the empirical data set was very good, with all five runs finding the same maximum likelihood parameter set for each model. The preferred model using both AIC or BIC was CS-DD with no anagenesis (four free parameters) (Table 2). The loglikelihood difference between the best CS model and the best IW model (both diversity-dependent) was 6.43. This value points to the CS model as the best model also in the likelihood ratio bootstrap test (see below). The models without anagenesis had virtually the same parameter values as their counterparts allowing anagenesis to be different from 0. That is, the latter models had estimated rates of anagenesis that were very close to 0. This is to be expected because all five frog clades radiated and hence there is no evidence of anagenesis. The only signal of anagenesis in such a case could come from the observation of recolonizations of the same mainland species that established the clade(s). This is because the model assumes that recolonizations can only occur after speciation has taken place (if it happens before speciation takes place, the recolonization is assumed to reset the colonization time and is then not observed). As the data did not contain recolonizations, the maximum likelihood estimate of anagenesis is expected to be 0. Across the 100 sets of empirical trees, the loglikelihood difference between the diversity-dependent CS and IW models ranged from 5.16 to 6.63, all of which suggest the CS model is highly preferred.

**Table 2.** Maximum likelihood parameter estimates and corresponding loglikelihood (LL) for five fitted models

model	$\lambda_{0}^{c}$	μ	K	γ	$\lambda^{a}$	LL	df	AIC	AIC weight	BIC	BIC weight
DI	0.18	0.03	∞	0.0002	0^*^	−215.87	4	439.75	0.00	445.53	0.00
DD-CS	0.44	0.11	36.44	0.0002	0^*^	−208.67	5	427.34	0.27	434.58	0.15
DD-CS-noA	0.44	0.11	36.45	0.0002	0	−208.67	4	425.34	0.73	431.13	0.85
DD-IW	0.40	0.17	131.89	0.0003	0^*^	−215.10	5	440.20	0.00	447.43	0.00
DD-IW-noA	0.40	0.17	131.96	0.0003	0	−215.10	4	438.20	0.00	443.98	0.00

^*^Indicates that the estimated value was numerically not exactly 0, but this is due to the stopping criterion of the optimization; it was always smaller than 10^−4^.

DI: diversity-independent rates; DD-CS: clade-specific diversity-dependence in colonization and cladogenesis; DD-CS-noA: same, but with anagenesis rate fixed to 0; DD-IW: island-wide diversity-dependence in colonization and cladogenesis; DD-IW-noA: same, but with anagenesis rate fixed to 0. df: degrees of freedom, i.e., number of free parameters; AIC: Akaike information criterion; BIC: Bayesian information criterion.

### Goodness-of-Fit

**Figure 3.**
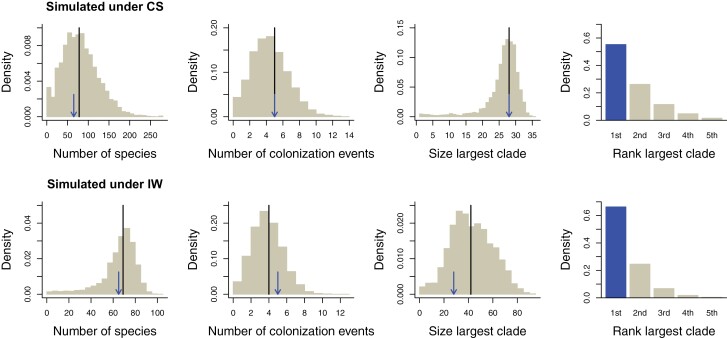
Goodness-of-fit plots. Distributions of relevant metrics (number of species, number of colonizations leading to extant clades, size of the largest clade, and rank of the largest clade when clades are ordered according to their colonization time, rank 1 corresponding to the first colonization) obtained from 5000 data sets simulated with the maximum likelihood parameters of the CS (top row) and IW (bottom row) models. Line (black in color version): median value; arrow and dark bar (blue in color version): value in the empirical data.

Using the estimated parameters for the diversity-dependent CS and IW models we generated simulated data for which we computed several summary statistics. The distributions of the summary statistics across these simulations fitted well with the empirical data for both models, but somewhat better for the CS model (Fig. 3; Supplementary Figs. S1 and S2), as the empirical statistics and the medians across the simulations are slightly more similar for this model.

### Bootstraps

The analyses fitting the CS model to each of 1000 CS and 1000 IW simulated data sets were all completed successfully. For the analyses fitting the IW model to the same data sets, some runs could not be completed within the limit we set (10 days). For the CS simulated data sets this was 0.8% of the simulations and for the IW simulated data sets this was 1.6%. These were all data sets with little information (only a single clade) where the estimation procedure went to very high values of the rate of cladogenesis and colonization. We used the loglikelihood that was obtained after 10 days which is thus an underestimate of the maximum IW loglikelihood, but the ML may not be much higher than this value after 10 days. However, even if we make the unlikely assumption that in all of these aberrant simulated data sets the IW model is a better fit, they are so rare that our qualitative conclusion that the CS model is a better fit does not change.

**Figure 4.**
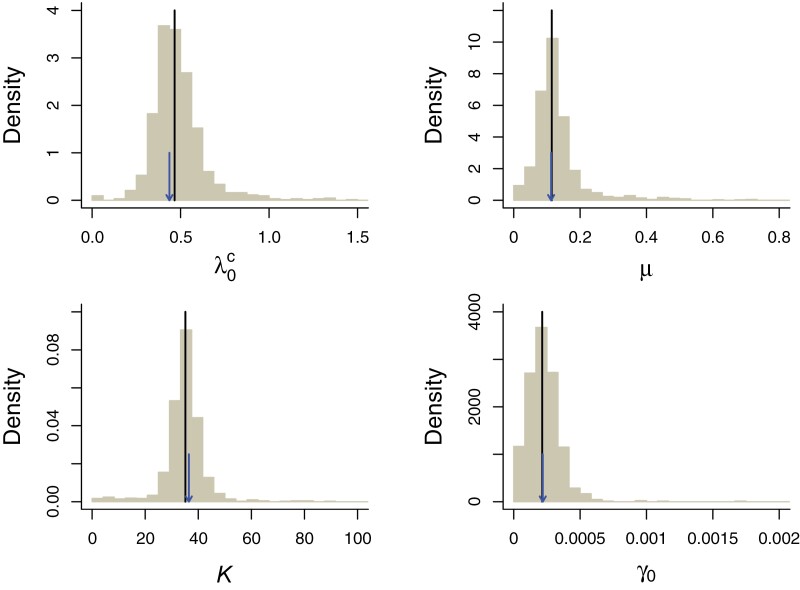
Bootstrap precision estimates of the parameters of the CS model. In a parametric bootstrap analysis the CS model was fitted to 1000 data sets simulated with the maximum likelihood parameters of the CS model for the empirical data. The panels show density histograms of the estimated parameters. The lines indicate the median estimated values across all simulations and the arrows point to the values used in the simulations.

**Figure 5.**
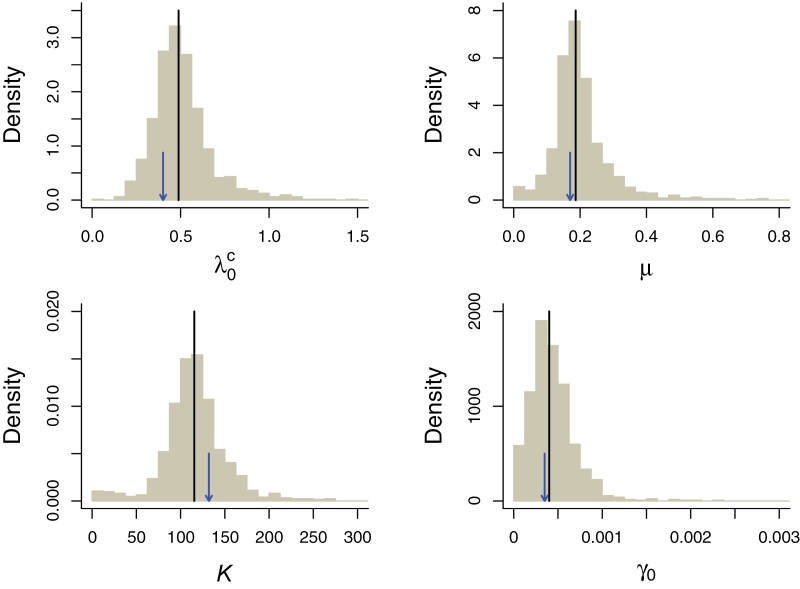
Bootstrap precision estimates of the parameters of the IW model. In a parametric bootstrap analysis the IW model was fitted to 1000 data sets simulated with the maximum likelihood parameters of the IW model for the empirical data. The panels show density histograms of the estimated parameters. The lines indicate the median estimated values across all simulations and the arrows point to the values used in the simulations.

Parameters were estimated with high precision and little bias under both models: the median and means of the distribution of parameters estimated under the CS and IW models for data sets simulated under those models closely matched the simulated values (Figs. 4 and 5). When fitting the CS model to IW simulations (Supplementary Fig. S3) we observe that the K is estimated to be much higher than in the CS simulations (Fig. 4). This is because the IW simulations show more variability in clade sizes that can only be accommodated by the CS model by assuming a larger clade-level K. When fitting the IW model to CS simulations (Supplementary Fig. S4), we do not observe such a discrepancy. Indeed, in this case the total number of species matters rather than the number of species per clade. All parameter estimates and corresponding loglikelihoods are available in Supplementary Table S3.

**Figure 6.**
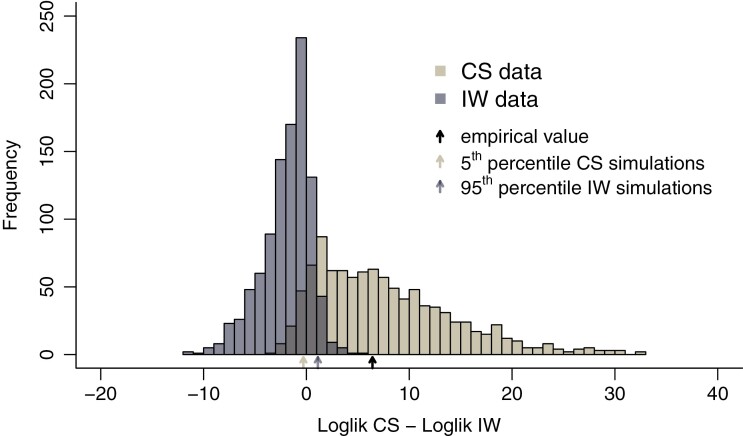
Likelihood ratio bootstrap test. Distribution of differences between the loglikelihood of the CS model and the loglikelihood of the IW model when fitting both models to data sets simulated under CS and IW. The rightmost arrow shows the difference in the empirical data, while the leftmost and middle arrows indicate the 5th percentile and the 95th percentile of the distributions generated under the CS model and the IW model respectively. The rightmost arrow falls well inside the distribution for data simulated under CS, and to the right of the 95th percentile (in fact even the maximum!) of the distribution for data generated under IW. Therefore, the CS model is strongly preferred.

The simulated data can be used to check the reliability of model selection (because we know the generating process). When performing model selection by simply selecting the model with the highest likelihood, the IW model was incorrectly preferred over CS in 7.7% of data sets simulated under CS. The CS model was incorrectly preferred over IW in 19% of data sets simulated under IW. When imposing at least two log-units of difference before selecting a model, these numbers become 0.9% and 1.6%, respectively. The CS and IW model were then correctly selected in 77% and 40.6% of the corresponding data sets respectively, leaving 22.1% and 57.8% undecided between the two models. Because using highest likelihood or higher by at least two log-units is quite arbitrary, and still leads to either high type I error (highest likelihood) or low power (two log-units difference), we used the bootstrap likelihood ratio (or loglikelihood difference) distribution to set the permissible type I error to 5% (two left-most arrows in Fig. 6). This distribution of differences in loglikelihood between the CS and the IW model revealed that it was highly unlikely (*P* < 0.00) that the empirical loglikelihood difference (6.43) would have been found if the underlying model was IW, because the loglikelihood difference found in the empirical data (black arrow in Fig. 6) falls clearly beyond the tail of the distribution of loglikelihood differences obtained from data simulated under IW (higher than the largest likelihood), but falls right in the middle of the distribution of differences for data simulated under CS (at the 49.6th percentile). This all suggests that the CS model is strongly supported as the best model for the empirical data.

The power to select the generating model is relatively high. The power to detect CS is 85% (part of the distribution generated under the CS model that is larger than the middle arrow in Fig. 6) whereas the power to detect IW is 72% (part of the distribution under the IW model that is smaller than the left-most arrow in Fig. 6). If the empirical data had had a loglikelihood ratio between −0.29 (the leftmost arrow in Fig. 6) and 1.11 (middle arrow in Fig. 6), model selection would have been indecisive.

## DISCUSSION

We have developed a method to determine, using phylogenetic data on island colonization and branching times, whether diversity-dependence in rates of colonization and speciation is limited to species within a clade, or extends to species from different clades, or whether the information in the data is too limited to make a clear call. In Hispaniolan *Eleutherodactylus* frogs we find that models including diversity-dependence outperform models without a negative feedback of diversity on colonization and speciation rates, suggesting that diversity limits play an important role. Diversity limits operating at the clade-specific level (i.e., species from different colonizing clades do not interact) predominate over limits at the island-wide level (i.e., species from different clades reduce each other’s rate of colonization and speciation), because the model with clade-specific diversity-dependence clearly outperformed the model with island-wide diversity-dependence.

Although *Eleutherodactylus* frogs show repeated patterns of evolution into the same set of ecotypes (Dugo-Cota et al., 2019), these results suggest that these ecotypes do not interfere with each other across clades. One could argue that Figure 2 already tells us this because the overlap in ecotypes and in ranges between the clades on Hispaniola is limited, and hence species do not seem to interact across clades. However, one can also explain this pattern as a *consequence* of interaction across clades, because under IW earlier clades block later ones from radiating into the same habitats (both ecologically and spatially). Our results do not support this explanation and lead us to conjecture that there has been sufficient (niche) space that IW diversity-dependence does not occur. Of course, this may change in time: if we wait millions of years, (niche) space may eventually become saturated, but currently there is no signal of IW diversity-dependence. In summary, present-day spatial distributions and ecological distributions into ecotypes cannot be taken as evidence that species from different clades do not interact, as these patterns may be a consequence of such interactions in the past. The approach we have taken in this paper is to infer such diversity-dependence from the phylogenetic branching pattern. We have shown that if IW diversity-dependence operates, we would often pick up its signal from the phylogenetic data. In our *Eleutherodactylus* frog example, we did not, as there is only 1% chance that the pattern we observed would be generated by an IW model (i.e., only 1 in 100 simulations of an IW model we would obtain a loglikelihood ratio between CS and IW models which is equal or higher than observed for the empirical data).

Our simulations were limited to the parameters estimated from the *Eleutherodactylus* frog data. To assess the more general ability of our approach to identify CS and IW when they are operating would require analyzing many more simulated data sets for a wide range of the parameters sets. This is currently computationally unfeasible, because the likelihood maximizations, although performed with highly optimized code, take quite a bit of time (at least a few hours per data set), which bars extensive simulation studies across a sizeable number of replicates. Instead, we suggest that researchers wishing to compare CS and IW models for their study system should fit these models to their data and take the estimated parameters to run simulations, just like we did here. This allows one to establish whether CS and IW models can be distinguished by plotting figures such as Figure 6. We have shown that it is important to do so, because model selection based solely on AIC may be biased (Etienne et al., 2016).

The CS model assumes the same carrying capacity K for each clade, which is a constraint to each clade’s size, and hence our model selection may be somewhat biased towards IW, which only limits the overall number of species by its K. Because the CS model nevertheless outperforms the IW model, this is not an issue for this study, and it may be indicative of a similar K among clades, which is in line with ecotype space limiting the number of species equally in each clade. Still, models with different K values for each clade could in principle be fitted to the data to confirm this. In practice, however, this is not really feasible, because we are already estimating four or five parameters, and there may not be enough information in a data set of this size to allow for more parameters to be reliably estimated.

We have only considered two models of diversity-dependence: one where diversity-dependence only applies to species within the same clade, and one where it also applies to species of other *Eleutherodactylus* clades establishing on an island. Various other models can be conceived. First, diversity-dependence might apply to *Anurans* species beyond *Eleutherodactylus* or include other amphibians or even non-amphibians. We have chosen the level of *Eleutherodactylus* species as it seems, arguably, the largest group where the assumptions of equal rates of colonization, speciation and extinction are not too strongly violated. Our results indicate that diversity-dependence does not extend to this scale. Second, diversity-dependence could also occur at a higher taxonomic level, i.e., the number of clades, rather than the number of species within them may be limiting further colonization or diversification. Third, the effect of phylogenetic relatedness may also differ for speciation, extinction and colonization. For instance, Pires et al. (2017) found that speciation is mostly affected by within-clade diversity-dependence, whereas extinction is mostly affected by between-clade diversity-dependence. Fourth, one could define phylogenetic limits in terms of actual phylogenetic distances so that we move from a within- and between-clade dichotomy to a more continuous spectrum where some phylogenetically related clades may interact, but more distantly related clades do not. There are no likelihood methods for such models yet. One may have to resort to simulation-based approaches such as Approximate Bayesian Computation (Janzen et al., 2015). These methods need to integrate all possible trajectories of the clades through time, which is not trivial because the space of these trajectories is extremely high-dimensional.

We assumed no diversity-dependence in extinction and anagenesis to focus on the effect on colonization and cladogenesis, and for model and computational simplicity. However, diversity-dependence in extinction and anagenesis is conceivable. For extinction one may assume higher extinction rates for higher diversity. We note that this causes a stronger pull-of-the-present in lineages-through-time plots contrary to what is commonly observed (Phillimore and Price, 2008; Etienne et al., 2012). However, such a pull-of-the-present may not be visible in empirical data, because we fail to account for incipient species (Etienne and Rosindell, 2012). Likelihood methods that incorporate both diversity-dependence and protracted speciation do not yet exist, however. Diversity-dependence in anagenesis is also conceivable, but it may be both negative and positive. High diversity can inhibit anagenesis by limiting the ecological space to evolve into. However, higher diversity might also mean that there has been greater selective pressure for a species to evolve away from the mainland sister (sub)species. Anagenesis can also occur through drift alone simply due to long-term isolation from the mainland, in which case a diversity effect on anagenesis seems unlikely. One might also argue that if local adaptation is the primary cause of anagenesis, competition with other species (and thus diversity-dependence in anagenesis) is unlikely to prevent anagenesis. In such a scenario the species will probably not establish at all (some adaptation seems necessary to survive in a new environment) which would be accounted for by diversity-dependence in the colonization rate.

One may wonder what it is in the branching pattern that allows for selecting one model over the other. A possible candidate is the rank of the largest clade. The IW model can be expected to have the first clade as the largest because later clades will be suffering from diversity-dependence and hence not be able to grow very large. However, we noticed that the first clade is also almost equally often the largest clade under the CS model (Fig. 3). Hence, a pattern we may put down to incumbency and interclade competition (Silvertown, 2004; Schenk et al., 2013) arises equally prominently under a model without interclade competition. Apparently, in our empirical example time since colonization is a more important determinant of the size of a clade than diversity-dependence. The IW or CS nature of the colonization and diversification process, and thus the presence or absence of priority effects at the macroevolutionary scale, is hidden in a more complex way in the phylogenetic branching pattern that is not easily picked up by simple summary statistics but is detected by our likelihood ratio test. We do note that the estimate of the island-wide carrying capacity K (132) is quite a bit larger than the number of species present on the island (66), suggesting that the island is still far from saturation under the IW model. Other systems may have a lower K and the effect of priority effects may be relatively stronger. It is an interesting avenue to study whether there is a relationship between the magnitude of these priority effects and the invasibility of islands, which may contribute to our understanding of biological invasions (Fraser et al., 2015).

The new IW model may also be applicable in other fields, such as epidemiology where it may serve as a tool in understanding the spread of an infectious disease, e.g., a virus, in island-like systems such as schools or hospitals. In such systems, there may be multiple sources of infections that spread through the local population and can be modelled as colonizations. The carrying capacity is the number of children or patients. The IW model would be the appropriate model if once the host is infected, it builds up immunity against all strains, thus hindering further colonization and diversification. This scenario is most likely if the colonizing strains are phylogenetically related. The CS model would be a better description if a host can be infected by multiple strains, but within each strain there is viral interference (see e.g., Ojosnegros et al. (2010)). This scenario is most likely if the colonizing strains are phylogenetically (and hence functionally) distinct.

The CS model implementation allows incomplete phylogenetic information: if the island species are recognized (including their endemicity status), but their colonization or branching times are not known, the model integrates over the possible colonization and branching times. The same principle can be applied to the IW model, but this is currently computationally unfeasible. Randomly inserting missing species to obtain a complete data set and then repeating the analysis for each sampled complete phylogeny, as we have done, is probably the most straightforward way to get an idea of the impact of phylogenetic uncertainty. This procedure can also be used to account for inherent uncertainty that exists in all phylogenetic trees, by sampling phylogenies from the Bayesian posterior of trees and applying the maximum likelihood procedure to estimate parameters. Incomplete knowledge due to failure to recognize incipient or cryptic species is a more fundamental problem that the field has not been able to address completely satisfactorily. There are models that can account for this, e.g., the protracted speciation model (Etienne and Rosindell, 2012; Etienne et al., 2014; Lambert et al., 2015; Hua et al., 2022), but the mathematical approach to compute the likelihood under this model seems incompatible with the approach used for diversity-dependence models (Etienne et al., 2012; Laudanno et al., 2019). Simulation-based approaches may be the only (long) way to a resolution (Richter et al., 2020).

We have provided a new model for island biogeography with diversity-dependent feedback on colonization and diversification occurring between all island species. Our implementation of this IW model has some computational limitations for islands with large numbers of colonizations, particularly if these are non-endemic, but typical insular data sets with a moderate number of colonizations or a high level of endemism (such as our *Eleutherodactylus* frogs) are perfectly feasible. Our single empirical example serves as an illustration to the empiricist on how to explore the phylogenetic limits of diversity limits to diversification from phylogenetic data alone despite the limitations of phylogenetic data (Losos, 2011). Although this example showed a clearly better fit of the CS model, future applications may reveal different and more nuanced impacts of clade competition on diversification at a wider range of phylogenetic scales. Further extension of our approach to allow integrating ecological data with phylogenetic data (Harmon et al., 2019), for instance to examine simultaneous diversity-dependence and trait-dependence of diversification, is an exciting but challenging next direction.

## SUPPLEMENTARY MATERIAL

Data available from the Dryad Digital Repository: http://dx.doi.org/10.5061/dryad.t1g1jwt3n.
